# Assessment of the hepatoprotective effect of developed lipid-polymer hybrid nanoparticles (LPHNPs) encapsulating naturally extracted β-Sitosterol against CCl_4_ induced hepatotoxicity in rats

**DOI:** 10.1038/s41598-019-56320-2

**Published:** 2019-12-24

**Authors:** Ebtsam M. Abdou, Marwa A. A. Fayed, Doaa Helal, Kawkab A. Ahmed

**Affiliations:** 1 Department of Pharmaceutics, National organization of Drug control and Research (NODCAR), Giza, Egypt; 2 Department of Pharmaceutics and Industrial Pharmacy, Faculty of Pharmacy, MTI University, Cairo, Egypt; 3 Department of Pharmacognosy, Faculty of Pharmacy, University of Sadat City, Sadat City, Egypt; 4 Department of Pharmaceutics and Industrial Pharmacy, Faculty of Pharmacy, El-Fayoum University, El-Fayoum, Egypt; 5 Department of Pathology, Faculty of Veterinary Medicine, Cairo University, Giza, 12211 Egypt

**Keywords:** Drug development, Preclinical research

## Abstract

The hepatoprotective effect of *β*-Sitosterol (BSS), a natural phytosterol, after being formulated into a suitable pharmaceutical drug delivery system has not been widely explored. BSS was isolated from *Centaurea pumilio L*., identified and formulated as lipid-polymer hybrid nanoparticles (LPHNPs) using the poly(D,L-lactide-co-glycolide) polymer and DSPE-PEG-2000 lipid in different ratios. The selected formulation, prepared with a lipid: polymer: drug ratio of 2:2:2, had an entrapment efficiency (EE%) of 94.42 ± 3.8, particle size of 181.5 ± 11.3 nm, poly dispersity index (PDI) of 0.223 ± 0.06, zeta potential of −37.34 ± 3.21 and the highest drug release after 24 h. The hepatoprotective effect of the formulation at two different doses against CCl_4_ induced hepatotoxicity was evaluated in rats. The results showed that the BSS-LPHNPs (400 mg/kg) have the ability to restore the liver enzymes (alanine aminotransferase (ALT) and aspartate aminotransferase (AST)), liver lipid peroxidation markers (malondialdehyde (MDA) and catalase (CAT)), total bilirubin and albumin to their normal levels without inhibitory effect on the CYP2E1 activity. Also, the formulation could maintain the normal histological structure of liver tissue and decrease the cleaved caspase-3 expression. LPHNPs formulation encapsulating natural BSS is a promising hepatoprotective drug delivery system.

## Introduction

Efficient oral absorption is sometimes hindered by different biobarriers which may be overcome by both polymer-based and lipid-based nanocarriers. Both liposomes and polymeric nanoparticles (NPs) are auspicious types of drug nanocarriers as they are characterized by being biocompatible and biodegradable^1^. Liposomes are known to have high degree of biocompatibility with accepted pharmacokinetic profile and the ability of being surface modified, especially with polyethylene glycol (PEG), resulting in prolonged circulation time^2^. Unfortunately, they have the disadvantages of being physically and chemically unstable as well as fast drug release rates and low entrapment efficiency^3^. Polymeric nanoparticles formulated by using either natural or synthetic polymers have gained great attention as they can be loaded with both water insoluble and water soluble drugs providing formulation with accepted stability and controlled drug release rates^4^. However, polymeric NPs have some drawbacks due to the use of toxic organic solvents during their preparation in addition to cytotoxicity and degradation of the polymer^5^.

Lipid-polymer hybrid nanoparticles (LPHNs) are now being developed to gather the advantages of both polymeric and lipid-based nanocarriers^6^. LPHNPs consist of a polymer core and a lipid shell resulting in shell-core structure^7^. With the appropriate selection of lipids and polymers, LPHNPs systems can demonstrate superior efficiency compared to that of their non-hybrid counterparts in improving the physicochemical properties of hybrid nanoparticles such as drug encapsulation, drug release modulation, physical stability improvement and cellular uptake enhancement^8–10^. Therefore, LPHNPs improve the oral delivery of challenging compounds, resulting in fewer side effects and enhanced patient compliance^11,12^.

The liver is a major organ that has a vital role in the elimination of xenobiotics from the body. Investigation of the hepatoprotective effect of natural herbal extracted drugs is very important especially because it is rarely to find synthetic drugs being used as effective hepatoprotectives. Carbon tetrachloride (CCl_4_) is an abundant hepatotoxin used for induction of hepatic cytotoxicity^13^ as it is metabolized in the liver releasing free radicals which results in lipid peroxidation and hepatocytes necrosis. A single exposure to CCl_4_ can lead to severe cetrizonal necrosis and steatosis that may be similar to symptoms of acute viral hepatitis^14^. In addition to necrosis, apoptosis which occurs in the ballooned and injured hepatocytes of the centrilobular area may coexist as an additional suggested mechanism for CCl_4_ toxicicity^15^.

*β*-Sitosterol (beta-sitosterol, (BSS) 24-ethylcholesterol) is a natural phytosterol that is a steroidal molecule similar to cholesterol but is of plant origin. It is present in many oils from plants and vegetables and is one of the most common dietary phytosterols that has been studied to have different pharmacological effects, such as amelioration of diabetes^16^, anticancer effects^17–20^, prevention of prostatauxe^21^, anxiolytic effects, and sedative effects^22^.

Regarding its effect on the liver, BSS containing diets can change the liver ultra-structure in both young and adult mice. BSS can also prevent gallstone formation and decrease serum and liver cholesterol, but only at high doses. No sufficient studies are found concerning the effect of BSS on different liver enzymes^23^. We found a few published papers in the literature regarding different pharmacological effects of plant extracts containing BSS and other phenols on the liver^24,25^. However, until now, there has been no study on the pharmacological effect of plant-extracted BSS in a suitable pharmaceutical formulation as a hepatoprotective agent against CCl_4_ induced toxicity in rats.

The aim of this study was to isolate and identify BSS from *Centaurea pumilio* L. (F: Asteraceae) plant and formulate it into LPHNPs using (1,2-distearoyl-sn-glycero-3-phosphoethanolamine-N-carboxy (polyethylene glycol)2000) (DSPE-PEG-2000) as a lipid and poly(D,L-lactide-co-glycolide) (PLGA) as a polymer. One selected formulation was evaluated for its hepatoprotective effect at two different doses against CCl_4_ induced hepatotoxicity in rats.

## Methods

### Materials

Poly (D,L-lactide-co-glycolide) (PLGA; lactide/glycolide ratio is 50:50, Mw = 40,000–75,000), (1,2-distearoyl-sn-glycero-3-phosphoethanolamine- N-carboxy (polyethylene glycol)2000) DSPE-PEG-2000, and Polyvinyl alcohol (PVA) were purchased from Sigma Chemical Co., USA. The 4-nitrocatechol and 4-nitrophenol were purchased from A Johnson Matthey Company (Royston, UK). Dichloromethane (DCM), methanol, ethyl acetate and chloroform were all of HPLC grade and were purchased from El-Gomhoria Co., Egypt. All other chemicals were of analytical grade and were purchased from El-Gomhoria Co., Egypt.

### Statement

All experiments and methods were performed in accordance with relevant guidelines and regulations. The protocol for the in- vivo studies was approved by Cairo University Institutional Animal Care and Use Committee ((CU- IACUC), Veterinary Medical and Agricultural Sciences Sector with the approval No. CU/II/F/11/19.

### BSS isolation from *Centaurea pumilio* L

#### Plant material

*Centaurea pumilio* L. (F: Asteraceae) aerial parts were collected in August, 2017 from Burg El-Arab (Egypt). The plant was kindly identified by Prof. Dr. A.A. Fayed, Professor of Plant Taxonomy, Faculty of Science, Assiut University, Assiut, Egypt. A voucher sample was kept in the Herbarium of the Faculty of Science, Assiut University, Assiut, Egypt.

#### Extraction and isolation

The dried and powdered aerial parts of *Centaurea pumilio* L. (1Kg) were sequentially extracted with *n*-hexane (3 × 1.5 L), DCM (3 × 1.5 L) and methanol (3 × 1.5 L). The extraction was performed in each solvent until exhaustion. The solvent was concentrated after completion of the extraction process under reduced pressure in a rotary evaporator (Heidolph, Germany) at 50 °C, yielding 5, 2 and 10 g for the n-hexane, DCM and methanol extracts respectively. The DCM extract (2 g) was subjected to column chromatography (2 × 100 cm) on silica gel (70–230 mesh) with gradient elution using DCM: ethyl acetate. Fractions eluted from the column were collected (20 mL each) and monitored using thin layer chromatoghraphy (TLC). Similar fractions were gathered and combined depending on the number and colour of the spots on the precoated silica gel plate and sprayed with 10% H_2_SO_4_. Three fractions eluted with chloroform: ethyl acetate (9:1) that were found to be similar were combined and concentrated, from which the compound (BSS) was crystallised and isolated in pure form.

#### Test for steroid (Salkowski’s reaction)

A few crystals of BSS were dissolved in DCM, and a few drops of concentrated H_2_SO_4_ were added to the solution. The upper DCM layer attained a reddish colour^26^.

#### Liebermann Burchard’s reaction

A few crystals of BSS were dissolved in DCM, and a few drops of concentrated H_2_SO_4_ were added, followed by the addition of 2‐3 drops of an acetic anhydride solution. The mixture turned violet blue and finally green^26^.

#### Test for alcohol

Four grams of ceric ammonium nitrate was dissolved in 10 mL of 2 N HNO_3_, on mild heating. A few crystals of BSS were dissolved in 0.5 mL of dioxane. The solution was added to 0.5 mL of the ceric ammonium nitrate reagent, diluted to 1 mL with dioxane and shaken well. The solution colour developed from yellow to red, indicating the presence of an alcoholic hydroxyl group^27^.

#### Spectroscopic characterization

Spectroscopic methods using infrared (IR), proton nuclear magnetic resonance (1 H‐NMR) and ultraviolet (UV) were used to elucidate the structure of the isolated compound (BSS). The infrared spectrum was recorded on a Shimadzu Infrared-400 spectrometer (Kyoto, Japan), the ^1^H‐NMR spectra were recorded using CDCl_3_ as the solvent on a Topspin (300 MHz, Bruker, Germany; from Cairo University), and the UV spectra were collected on a Shimadzu (1601 UV/VIS) spectrophotometer.

#### HPLC determination of BSS

BSS was determined by the HPLC method previously developed by Lee *et al*.^28^. An HPLC system (Shimadzu, Tokyo, Japan) was used. The mobile phase was methanol: acetonitrile (the gradient solvent system was initially 30:70, increased in a linear gradient to 30:70 for 20 min, 0:100 for 10 min, and finally 30:70 for 15 min), with λmax = 210 nm and a flow rate of 1 mL/min. The system consisted of a UV detector and a manual injector with a 20-µL loop, and the column used was SunFire C-18 stainless steel (2.1 mm × 50 mm, 5 mm; Waters Corporation, Milford, MA, USA). All samples were filtered and degassed just before use.

A stock solution of BSS was prepared by transferring 100 mg to a volumetric flask and dissolving in 100 mL acetonitrile to give a final concentration of 1 mg/mL. Then, serial dilutions were done and the calibration curve was constructed.

#### Preparation of BSS-LPHNPs

BSS-LPHNPs were prepared using a previously reported single-step nanoprecipitation method^29,30^. BSS and PLGA were dissolved in 5 mL of DCM in a beaker as the organic phase. DSPE-PEG-2000 was dissolved in 5 mL ethanol, which was then dispersed into a solution of 1.5% (w/v) PVA as the aqueous phase. The organic phase was added drop wise into the aqueous phase with continuous stirring at 1000 rpm. The mixture was stirred at room temperature until the organic solvent was totally evaporated. The dispersion was then subjected to sonication using a probe sonicator (Sonifier^®^ 250 Branson, USA) in ice bath (4 °C) for each cycle of 5 min, resting for 5 min between cycles to avoid excessive heat generation that may lead to product degradation^31^. The suspension was centrifuged using a cooling centrifuge (Remi Laboratory Centrifuge R32A, Remi Equipment, Bombay, India) at 10000 rpm for 15 min. The supernatant was separated, and the solid particles were stored in tightly closed glass vials at 4 °C. Six formulations were prepared using different lipid: polymer: drug ratios as indicated in Table 1.

**Table 1:** BSS-LPHNPs formulations composition and physicochemical evaluation.

BSS-LPHNPs formulations	DSPE-PEG2000: PLGA: BSS	EE%*	Particle size (nm)*	PDI*	Zeta potential*	% BSS release after 12 h^†^	% BSS release after 24 h^†^
F1	1:2:1	35.24 ± 4.57	251.5 ± 14.57	0.343 ± 0.09	−19.23 ± 3.47	20.51 ± 2.87	27.51 ± 2.71
F2	2:2:1	45.62 ± 4.5	154.8 ± 12.6	0.321 ± 0.07	−21.53 ± 2.34	24.57 ± 3.23	37.51 ± 2.85
F3	3:2:1	39.57 ± 3.67	124.2 ± 8.4	0.308 ± 0.11	−23.43 ± 4.51	35.74 ± 2.86	47.72 ± 3.63
F4	1:2:2	61.57 ± 3.6	269.7 ± 22.4	0.284 ± 0.08	−33.72 ± 4.05	55.42 ± 3.02	71.46 ± 2.72
F5	2:2:2	94.42 ± 3.8	181.5 ± 11.3	0.223 ± 0.06	−37.34 ± 3.21	86.56 ± 2.63	92.57 ± 5.85
F6	3:2:2	70.21 ± 2.7	156.8 ± 9.3	0.215 ± 0.12	-40.24 ± 4.31	75.37 ± 2.57	83.42 ± 2.27

*Results are expressed as mean ± SD (n = 3)

^†^Results are expressed as mean ± SD (n = 6).

#### Evaluation of the prepared BSS-LPHNPs

*Entrapment efficiency (EE %)*. Determined amounts of the prepared nanoparticles were dispersed into 5 mL DCM under high-speed stirring. The dispersion was then centrifuged at 10000 rpm at 4 °C until a clear supernatant was obtained. The clear supernatant was filtered using a 0.45 membrane filter (PVDF, Millipore, County Cork, Ireland), and a volume of 20 µL was injected into an HPLC system. BSS concentration was determined and EE % was calculated using the following equation:

$${\rm{EE}} \% =\frac{{\rm{Entraped}}\,{\rm{amount}}\,{\rm{of}}\,{\rm{BSS}}\times 100}{{\rm{Initial}}\,{\rm{amount}}\,{\rm{of}}\,{\rm{BSS}}}$$

#### Particle size analysis

Particle size analysis (mean diameter and polydispersity index (PDI)) of the prepared BSS-LPHNPs was performed using dynamic light scattering (Zeta-sizer Nano ZS-90, Malvern Instruments, Worcestershire, UK) after appropriate dilution with distilled deionized water. Standard operation procedures were used for controlling all the measurements and analysis setting. All measurements were carried out in triplicate and the mean and SD values were calculated.

*Measurement of zeta potential*. Zeta potential of the prepared nanoparticles formulations as well as their charges were investigated using a Zeta-sizer (Zeta-sizer Nano ZS-90, Malvern Instruments, Worcestershire, UK) under standard operation conditions. Samples were appropriately diluted with distilled deionized water. The results were expressed as the mean values (n=3) ± SD.

*In-vitro drug release study*. The BSS release profile from different prepared BSS-LPHNPs was determined using the dialysis tube diffusion method^32^. Determined amount of the BSS-LPHNPs was suspended in 1 mL phosphate-buffered saline (PBS pH 7.4) and added in the dialysis tube (M.Wt. cut off: 500–1000. Medicell, London, UK), both ends of the tube were tightened. The tube was tied to the paddle of a USP dissolution apparatus I (USP dissolution tester apparatus, SR8 Plus, Hanson Research, USA) and immersed into a vessel containing 900 mL of PBS (pH 7.4) at 37 ± 0.5 °C with constant stirring at 100 rpm. Dimethylsulfoxide (DMSO: 1%) was added into the PBS to impart the sink conditions^33^. The samples (0.5 mL) were taken at different time intervals (0.5, 1, 2, 3, 6, 12, 24, 36 and 48 h) and replaced with fresh pre-heated medium. The samples were analyzed for BSS content, cumulative drug release was calculated and plotted against time. Each experiment was performed six times and average values were taken.

#### Transmission electron microscopy (TEM)

The prepared BSS-LPHNPs formulation (F5) was microscopically examined using a TEM (Joel JEM 1230, Tokyo, Japan). The samples were prepared by dispersing into double deionized water. One drop was placed onto a carbon-coated copper grid, and the excess was drawn off. The samples were left to dry for 5 min, and TEM images were taken.

#### Stability studies

Stability studies were performed for the selected LPHNPs formulation (F5) to investigate any physicochemical properties changes during storage. The NPs, dispersed into 1 mL PBS and kept in tightly closed glass vials, were subjected to accelerated stability studies as per International Council for Harmonisation (ICH) guidelines as they were stored at refrigerator temperature (4 °C/60 ± 5% RH) and at room temperature (25 °C/60 ± 5% RH)^34^. At different time intervals (7, 15, 30, 60, and 90 days), the samples were withdrawn, examined using a polarized light microscope (JEM-100S, Jeol Ltd.,Japan), and analyzed for particle size, PDI, zeta potential, and EE %^35^.

#### *In- vivo* studies

Animals: Adult male Sprague–Dawley rats weighing 250–270 g were obtained from the breeding colony maintained at the animal house of the National Organization for Drug Control and Research (NODCAR, Giza, Egypt). The animals were allowed free access to a standard diet and tap water ad libitum and were housed at room temperature under natural light/dark conditions for 1 week before starting the experiments.

### Experimental design

The rats (n = 48) were randomly divided into six groups with 8 rats in each group. The following protocol was followed:

**Group 1**: The normal group.

**Group 2**: The rats received saline and Tween 20 (1% v/v).

**Group 3**: The rats were treated with free BSS suspended in saline and Tween 20 (1% v/v) at a dose level of 400 mg/kg body weight, once per day p.o., for 7 days via gastric intubation.

**Group 4:** The rats were treated with un-medicated LPHNPs (prepared using the same method as that for BSS-LPHNPs (F5) but without drug loading) suspended in Tween 20 (1% v/v in saline).

**Group 5**: The rats were treated with BSS-LPHNPs (F5) suspended in Tween 20 (1% v/v) at a dose of 200 mg/kg body weight, per day p.o., for 7 days.

**Group 6**: The rats were treated with BSS-LPHNPs (F5) suspended in Tween 20 (1% v/v) at a dose of 400 mg/kg body weight, per day p.o., for 7 days.

On the seventh day, a single dose of a mixture of CCl_4_ and olive oil (1:1) was given (50% v/v, 2 mL/kg, i.p.)^36^ to all animals except those in the normal group.

On the eighth day, exactly 24 h after the CCl_4_ injection, all the animals were sacrificed by head disclosure. The blood was collected, and the sera were separated. Serum alanine aminotransferase (ALT) and aspartate aminotransferase (AST) levels were estimated using laboratory kits (Diamond Diagnostics, Cairo, Egypt). Stanbio Laboratory kits (Stanbio Inc., Boerne, TX) were used to determine the serum total protein and albumin levels. All procedures were performed according to the instructions from the manufacturer. The catalase (CAT) levels in the serum samples of all the experimental animals were determined using Aebi’s method at 230 nm^37^. The level of lipid peroxidation was estimated by measuring malondialdehyde (MDA) levels using a previously described method^38^.

### Measurement of CYP2E1 enzyme activity

Eighteen adult male Sprague–Dawley rats were divided into three groups, two groups were used as positive and negative controls with no treatments and the third group received BSS-LPHNPs (F5) suspended in Tween 20 (1% v/v) at a dose of 400 mg/kg body weight, per day p.o., for 7 days. After the 7 days, all the animals were sacrificed by head disclosure. Liver was removed promptly, washed with cold physiological saline solution and cut into very small pieces. Liver microsomes were prepared as prescribed previously^39,40^. The CYP2E1 activity was measured by measuring the transformation of p-nitrophenol (PNP) to p-nitrocatechol with the isolated hepatic microsomes by a previously described method^41^. Liver microsomes were incubated into 0.1 M Phosphate buffer, pH7.2 containing 0.2 mM PNP (probe substrate) in an Eppendorf tubes to a final volume of 200 µL. For liver microsomes from the positive control group, 0.02 mM chlormethiazole was added to the incubation system for 5 min at 37 °C as a CYP2E1 inhibitor^42^.

The reaction was initiated by addition of NADPH (nicotinamide adenine dinucleotide phosphate) (1 mM). The mixture was incubated for 20 min at 37 °C then the reaction was terminated by addition of 1% TCA (Trichloroacetic acid). The resultant mixture was centrifuged at 5000 g for 10 min and 10 µL N NaOH was added to the supernatant. The resultant pink-yellow p-nitrocatechol absorbance was measured spectrophotometerically within few minutes at 510 nm. The concentration of p-nitrocatechol was determined from the standard calibration curve obtained by zero-time controls (which were obtained by the same procedure with the omission of liver microsomes, or PNP or NADPH or addition of TCA before NADPH). CYP2E1 activity was expressed as nmol p-nitrocatechol /min/mg microsomal protein.

### Histopathological examination of liver tissues

The specimens from the livers of all rats from the *in-vivo* studies were collected, fixed in 10% neutral buffered formalin, washed, dehydrated, cleared and embedded in paraffin. The paraffin blocks were sectioned at 4–5 micron thickness and stained with haematoxylin and eosin for histopathological examination^43^. Ten microscopic fields per section/rat were examined by a light microscope (Olympus BX50, Japan) under x200 & x400 magnification. The histopathological alterations were scored and graded, where (0) indicated no changes, and (1), (2) and (3) indicated mild, moderate and severe changes, respectively. The percentage grading was determined by percentage as follows: (<30%) showed mild changes, (<30–50%) indicated moderate changes, and changes more than 50% (>50%) indicated severe changes^44^.

### Immunohistochemical analysis

Apoptosis was determined by immunohistochemical analysis of the cleaved form of caspase-3 in liver sections as described by Eckle *et al*.^45^. Sections were stained with rabbit-anti-cleaved caspase-3 (1:100 dilution) (Cell Signaling, Danvers, MA, USA). The immune reaction was visualized using diaminobenzidine tetrachloride (DAB, Sigma Chemical Co., St. Louis, MO, USA). Staining intensity and its distribution were graded as negative (no staining), weak, moderate, or strong intensity. Quantification of cleaved caspase-3 apoptotic protein was estimated by measuring the area % expression from 5 randomly chosen fields in each section and averaged using image analysis software (Image J, version 1.46a, NIH, Bethesda, MD, USA).

### Statistical analysis

The data from all experiments were expressed as the mean value ± SD. The statistical data were analyzed by one-way analysis of variance (ANOVA), and p < 0.05 was considered to be significant with 95% confidence intervals. The data of liver enzymes and functions were expressed as the mean ± standard error of the means (SEM), n = 6. Statistical analysis was carried out using one-way ANOVA followed by the Tukey–Kramer multiple comparison test. Probability values of less than 0.05 were considered statistically significant whereas the graphs were drawn using Prism computer program (GraphPad software Inc., V5, San Diego, CA).

## Results

### BSS isolation and identification

BSS is a colorless crystalline compound, λ_max_ in CHCl_3_: 220 nm. The IR spectrum revealed several bands with absorption at 3380 cm^−1^ for stretching of OH group, 2920 cm^−1^ for aliphatic CH stretching, 1639 cm^−1^ for stretching of C = C, and 1451, 1362, 1066 and 1016 cm^−1^ for geminal dimethyl bending^1^. HNMR (CDCl_3_, 300 MHz) gave characteristic peaks at δ_H_ 3.21 (1 H, m, H-3), 5.28 (1 H, m, H-6), 5.16 (1 H, m, H-23), 4.69 (1 H, m, H-22), 3.65 (1 H, m, H-3), 2.40 (1 H, m, H-20), 1.69–2.03 (5 H, m) ppm. Several peaks detected at δ_H_ 0.79–0.95 (m, 9 H), 0.99–1.08 (m, 5 H), 1.37–1.45 (m, 4 H), 0.68–0.71 (m, 3 H), 1.80–2.05 (m, 5 H), 1.08–1.14 (m, 3 H), 1.36–1.64 (m, 9 H) ppm.

Upon inspection of the IR spectrum, showed bands at 3380 cm^−1^, representing O-H stretching, another one at 2920 cm^−1^ is due to aliphatic or C-H stretching or (CH_3_), in addition to a band at 1639 cm^−1^ is assigned for double bond stretching of (C=C), and one at 1016 cm^−1^ is relative to (C-O). The ^1^HNMR (300 MHz, CDCl_3_) of the compound reveals a one-proton multiplet at δ_H_ 2.40, which indicates 3 H representing a steroidal nucleus. The 6 protons typically constituting the steroidal nucleus appear as a multiplet at δ_H_ 5.28 that corresponds to a single proton. In addition the spectrum also revealed other peaks at δ_H_ 1.45 and δ_H_ 1.14 each representing 3 protons assigned for a pair of tertiary methyl groups corresponding to two carbons; number 18 & 19 respectively. The ^1^HNMR possessed another two doublets present at δ_H_ 0.91 (*J* = 6.7 Hz) and δ 0.88 (*J* = 6.7 Hz) assigned for two methyls attached to C-26 and C-27, respectively. While there was a doublet present at δ_H_ 1.60 (*J* = 6.5 Hz) represented a methyl group at C-21. In addition, a triplet representing three hydrogen atoms δ_H_ 0.89 representing a methyl at C-29. This compound consists of six methyls, eleven methylene groups in addition to three quaternary carbons and a hydroxyl. All the previous analytical data are very closely related to the data reported in the literature for *β* – Sitosterol^46,47^.

### Evaluation of the prepared BSS-LPHNPs formulations

#### Entrapment efficiency (EE%)

The EE% values of the prepared BSS-LPHNPs are shown in Table 1. The PLGA polymer was used at a constant ratio in all formulations. An increase in the lipid ratio from 1 to 2, either in the presence of lower or higher amounts of BSS, significantly (p < 0.05) increased the EE% (from 35.24 ± 4.57 to 45.62 ± 4.5 for LPHNPs (F1) and LPHNPs (F2) respectively and from 61.57 ± 3.6 to 94.42 ± 3.8 for LPHNPs (F4) and LPHNPs (F5) respectively). A further increase in the lipid ratio significantly (p < 0.05) decreased the EE% for the formulation prepared by low drug loading, LPHNPs (F3), and that with high drug loading, LPHNPs (F6); the EE% values were 61.57 ± 3.6 and 70.21 ± 2.7 for F3 and F6, respectively. An increase in the BSS ratio from 1 to 2 significantly (p < 0.05) increased the EE% at the same lipid ratio.

#### Particle size, PDI, and zeta potential

The particle size, PDI and zeta potential values of the prepared LPHNPs are collected in Table 1. The particle size of the prepared NPs was affected by both lipid and drug ratio. There was a significant particle size decrease with DSPE-PEG-2000 ratio increase at either a low or high drug ratio. On the other side, there was a significant increase of the particle size with the drug ratio increase from 1 to 2 at the corresponding lipid ratio except for LPHNPs (F1) and (F4). These two formulations, having the lowest lipid ratio, had the highest particle size with no significant difference between each other (P value = 0.583). As the particle size, the increase of the lipid ratio decreased the PDI of the NPs. However, contrary to the particle size, increase of the drug ratio significantly (p < 0.05) decreased the PDI at the same lipid ratio. The zeta potential of BSS-LPHNPs had relatively high negative values due to the presence of the negatively charged lipid, DSPE-PEG-2000, on the particles surface with an increase in the zeta potential value along with an increase in the lipid amount.

#### In- vitro drug release

**Figure 1:**
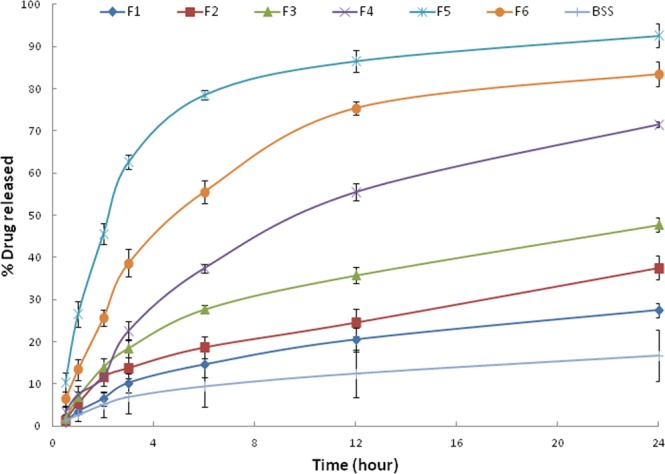
*In-vitro* BSS release from the prepared BSS-LPHNPs formulations.

The release profiles of BSS from the prepared NPs are represented in Fig. 1, and the values of the % release after 12 and 24 h are collected in Table 1. The formulations prepared at the higher BSS ratio, F4, F5 and F6 had faster and higher drug release than those prepared at the smaller BSS ratio. LPHNPs (F5), having the highest EE%, had fast drug release ratio of 86.56 ± 2.63 and 92.57 ± 2.85 at 12 and 24 h, respectively. This formulation (F5) had the highest EE% and acceptable particle size, PDI and zeta potential values, so it was selected for further evaluation.

#### TEM morphology

**Figure 2:**
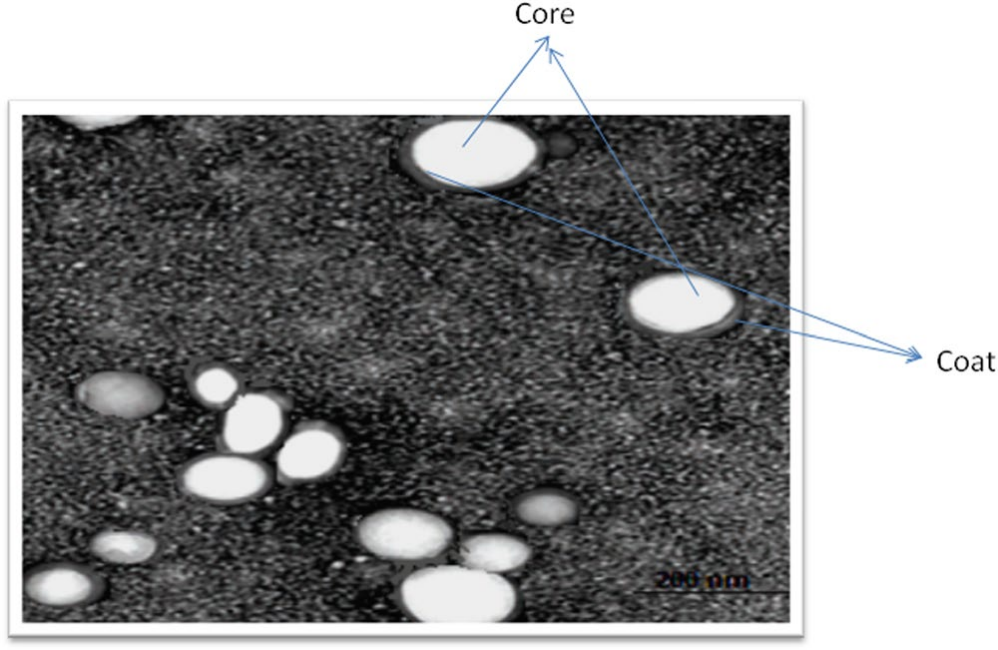
TEM micro-photo of the prepared BSS-LPHNPs (F5).

Transmission electron microscopy (TEM) was utilized to study the morphology of the selected BSS-LPHNPs formulation (F5), Fig. 2. The BSS-loaded LPHNPs appearance ranged from round to oval in shape with smooth surfaces, bright internal core which may indicate presence of the polymeric core in which the drug is encapsulated and dark shell indicating presence of the lipid layer which is in accordance with previous studies^48,49^.

#### Stability studies

The stability study results of the LPHNPs (F5) are shown in Table 2. The NPs were stored under two different storage conditions. For NPs stored at 25 °C/60 ± 5% RH, starting from day 15, there was a significant decrease in the EE% and zeta potential with a corresponding increase in the particle size and PDI, indicating leaching of the drug from the prepared LPHNPs with formation of small aggregations having larger size and PDI. The storage effect at these conditions increased with time with formation of large aggregations, which indicates that 25 °C/60 ± 5% RH aren’t the optimized conditions for BSS-LPHNPs storage. On the other hand, upon storage of the NPs at 4 °C/60 ± 5% RH, there were a significant EE% and zeta potential decrease and a significant particle size and PDI increase only after 90 days of storage, indicating that these conditions are suitable for BSS-LPHNPs storage up to 3 months which is in accordance with previous results^36,50^.

**Table 2:** stability study of the selected BSS-LPHNPs formulation (F5).

Time (days)	Microscopic observation (presence of aggregations)	Encapsulation efficiency%	Particle size (nm)	PDI	Zeta potential (mV)
**At 25 °C/60 ± 5% RH**					
0	No	94.42 ± 3.8	181.5 ± 11.3	0.223 ± 0.06	−37.34 ± 3.21
7	No	93.64 ± 2.54	208.5 ± 9.7	0.227 ± 0.09	−36.42 ± 3.57
15	yes	90.72 ± 2.15	246.4 ± 10.2	0.292 ± 0.12	−32.45 ± 4.72
30	yes	83.65 ± 3.12	293.3 ± 8.7	0.354 ± 0.15	−27.64 ± 5.24
60	yes	75.63 ± 3.25	364.8 ± 13.4	0.414 ± 0.19	−21.57 ± 3.95
90	yes	69.73 ± 4.32	446.7 ± 12.6	0.502 ± 0.15	−18.63 ± 4.56
**At 4 °C/60 ± 5% RH**					
0	No	94.42 ± 3.8	181.5 ± 11.3	0.223 ± 0.06	−37.34 ± 3.21
7	No	94.12 ± 2.83	192.7 ± 7.9	0.226 ± 0.08	−37.64 ± 4.21
15	No	93.84 ± 2.81	206.3 ± 10.2	0.235 ± 0.08	−36.34 ± 3.54
30	No	93.53 ± 3.13	212.5 ± 9.7	0.252 ± 0.11	−33.45 ± 4.23
60	No	91.54 ± 3.42	230.4 ± 12.4	0.307 ± 0.14	−29.62 ± 3.87
90	yes	89.56 ± 4.56	242.3 ± 11.7	0.312 ± 0.11	−26.05 ± 4.15

*Results are expressed as mean ± SD (n = 3).

### In- vivo studies

**Figure 3:**
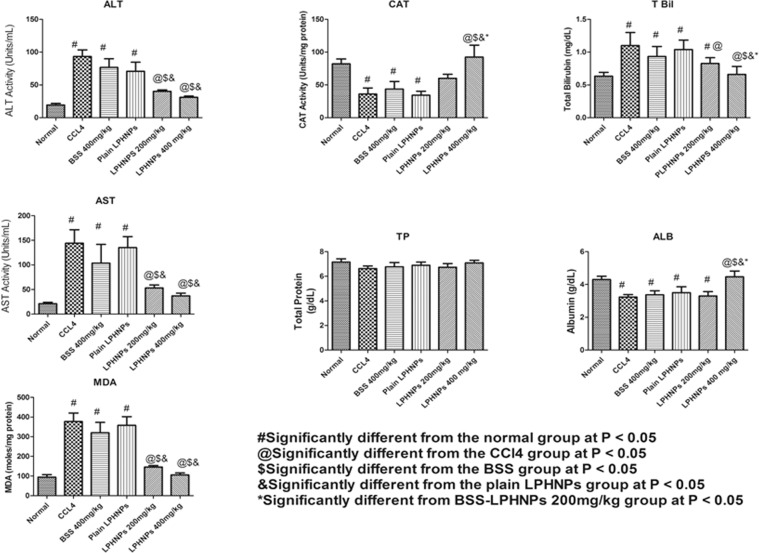
Effect of BSS-LPHNPs on hepatic functional enzymes and hepatic lipid profile after CCl_4_-induced hepatotoxicity.

The results of the hepatoprotective effect of the BSS-LPHNPs at two different doses on the rats treated with CCl_4_ are represented in Fig. 3. Hepatotoxicity was induced using CCl_4_ which is known to induce hepatic damage through different mechanisms such as decreasing the activity of antioxidant enzymes, formation of free radicals as well as lipid peroxidation^51^.

### Effect on the serum levels of ALT and AST

The results showed that injection of CCl_4_ significantly increased the serum levels of both ALT and AST compared to those of the normal rats. Rats pre-treated with either a BSS suspension or plain LPHNPs had slightly decreased the ALT and AST levels but were still not significantly different from the CCl_4_ group, indicating the in-ability of these two treatments to protect the liver from the hepatotoxic effect of CCl_4_. However, BSS-LPHNPs, at both doses of 200 and 400 mg/kg, succeeded maintaining ALT and AST at non-significant levels compared to those of the normal group. Thus, BSS-LPHNPs could resist the hepatotoxic effect of CCl_4_ on the ALT and AST levels at either low or high dose, with a non-significant difference between both doses.

### Effect on MDA and CAT (lipid peroxidation effect)

CCl_4_ injection in rats significantly increased the MDA levels and decreased the CAT levels compared to rats of the normal group. BSS-LPHNPs pre-treatment at both doses resulted in lowered MDA levels, which weren’t significantly different from rats of the normal group. In contrast, other treatments, failed to maintain the MDA levels at normal. In the case of CAT, only BSS-LPHNPs at a high dose (400 mg/kg) succeeded maintaining normal levels of CAT after CCl_4_ injection.

### Effect on total bilirubin (T. Bil)

CCl_4_ injection into the rats significantly increased T. Bil level. Pre-treatments of the rats with either a BSS suspension or plain LPHNPs failed to protect T. Bil levels from being elevated after CCl_4_ injection. Pre-treatment with BSS-LPHNPs at the lower dose (200 mg/kg) resulted in a T. Bil level that was significantly lower than that of the CCl_4_ group but still higher than the normal levels. Only pre-treatment with BSS-LPHNPs at the 400 mg/kg dose maintained T. Bil at a normal level after injection with CCl_4_.

### Effect on albumine (ALB) and total protein (TP)

Only BSS-LPHNPs at the high dose (400 mg/kg) could restore the ALB levels to normal after the latter were decreased significantly (p value < 0.05) by the CCl_4_ injection. In terms of the effect on TP, there was no significant difference between all groups, as CCl_4_ didn’t significantly affect the TP levels.

### CYP2E1 enzyme activity

CYP2E1 enzyme activity of control groups (positive and negative) and BSS-LPHNPs (400 mg/Kg) is represented in Table 3. Non-significant difference of the enzyme activity was found between the all groups at the base line. Non-significant difference of the enzyme activity was found between the base line and after 7 days for the negative control group (which did not receive any treatment) and BSS-LPHNPs group indicating absence of inhibitory effect of BSS-LPHNPs on the CYP2E1 enzyme activity. On the other hand, significant difference was found between the base line and after 7 days for the positive control group which was treated by the enzyme inhibitor, chlormethiazole, indicating significance of the used assay method.

**Table 3:** CYP2E1 enzyme activity (nmol p-nitrocatechol /min/mg microsomal protein).

	Control group (Negative)	Control group (Positive)	BSS-LPHNPs (400 mg/Kg) group
At the base-line	3.04 ± 0.593	2.95 ± 0.631	3.15 ± 0.638
After 7 days	2.86 ± 0.764	0.806 ± 0.212	3.31 ± 0.827

Results are expressed as mean ± SD (n = 6).

### Histopathological studies

**Figure 4:**
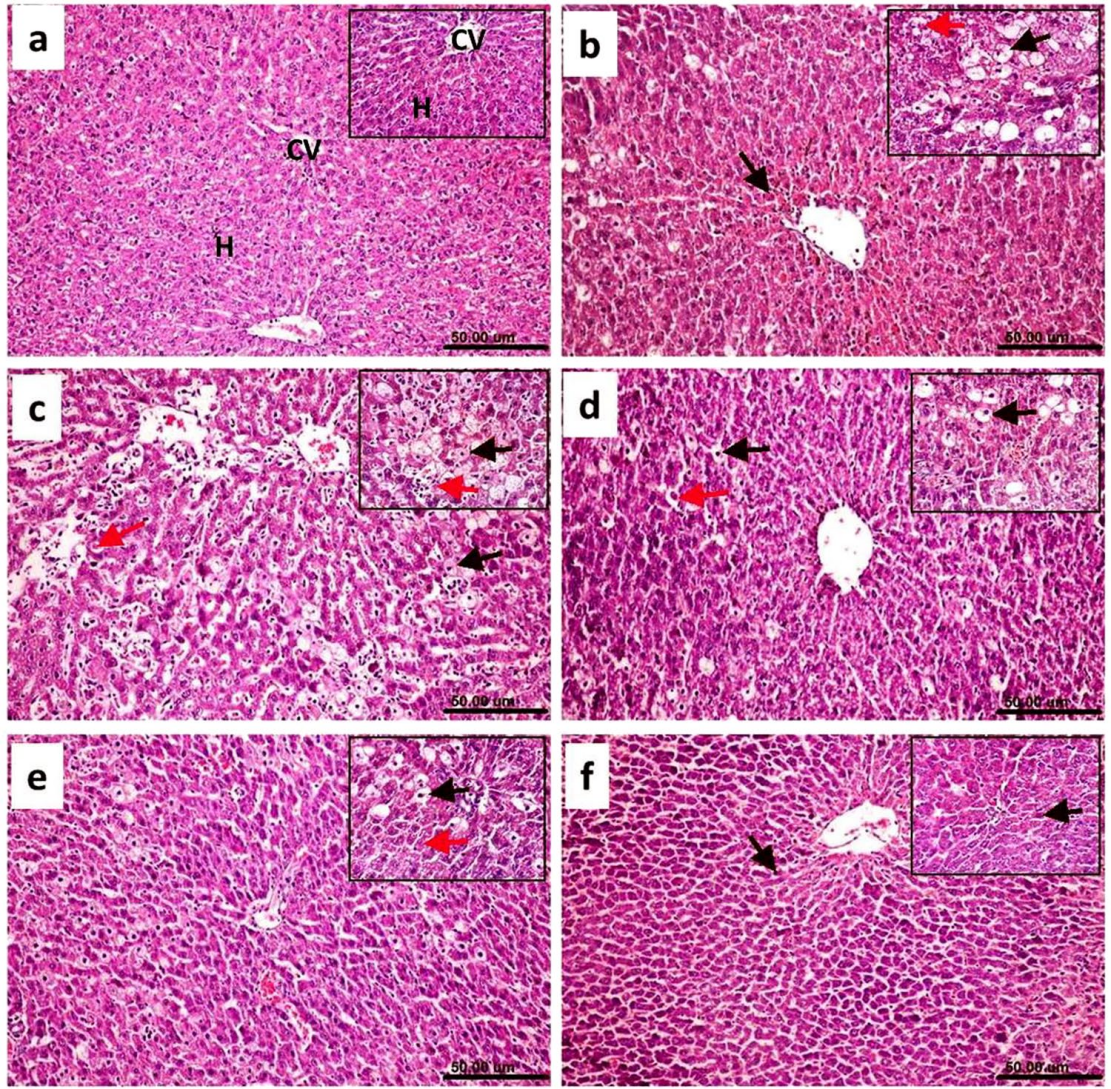
Liver of rat (**a**) from normal group showing the normal histological structure of hepatic lobule. Note normal central vein (CV) and normal hepatocytes (H). (**b**) Treated with CCl4 showing massive centrilobular hepatocellular necrosis associated with haemorrhage and mononuclear inflammatory cells infiltration (arrow), insert image showing ballooning degeneration of hepatocytes with pyknosis of their nuclei (black arrow) and apoptosis of hepatocytes (red arrow). (**c**) Co-treated with CCl4 + BSS suspension showing ballooning degeneration of hepatocytes with pyknosis of their nuclei (black arrow), apoptosis of hepatocytes (red arrow) and mononuclear inflammatory cells infiltration. (**d**) Co-treated with CCl4 + plain LPHNPs formula, showing ballooning degeneration of hepatocytes (black arrow) and apoptosis (red arrow). (**e**) Co-treated with CCl4 + BSS-LPHNPs (200 mg/kg), showing ballooning degeneration of some hepatocytes (black arrow) and cytoplasmic vacuolization and microvesicular steatosis of other hepatocytes (red arrow), (**f**) co-treated with CCl4 + BSS-LPHNPs (400 mg/kg) showing necrosis of sporadic hepatocytes (long arrow). (H&E, scale bar 50um, X200) (inserted images X 400).

**Figure 5:**
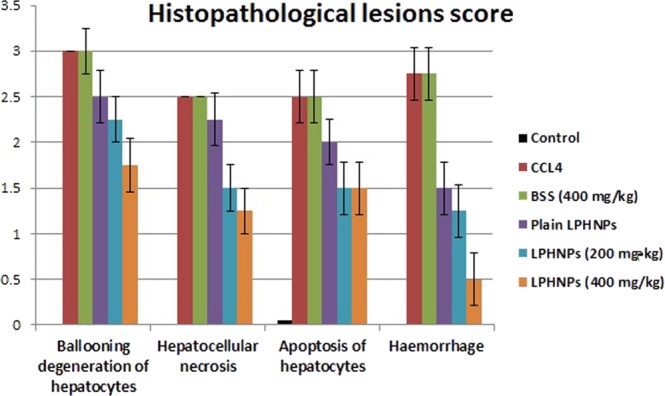
Histopathological lesions score in different groups. Data shown as mean ± SE; error bars show the variations of determinations in terms of standard error.

The microscopy images of the liver of the control normal rats indicated a normal histological structure of the hepatic lobule, in which the normal central vein and normal hepatocytes were arranged in hepatic cords around the central vein (Fig. 4a). In contrast, the liver of rats treated with CCl_4_ revealed severe histopathological alterations described as massive centrilobular hepatocellular necrosis associated with hemorrhage and mono nuclear inflammatory cell infiltration. Ballooning degeneration of hepatocytes, pyknosis of hepatocytic nuclei and apoptosis of hepatocytes were also noted (Fig. 4b) in all examined sections. Moreover, the livers of rats co-treated with CCl_4_ + BSS suspension revealed the same previously mentioned histopathological alterations. The examined sections from this group showed multiple focal hepatocellular necrosis, haemorrhage, ballooning degeneration of hepatocytes with pyknosis of their nuclei, apoptosis of hepatocytes and mononuclear inflammatory cell infiltration (Fig. 4c). Microscopic examination of the liver sections from rats co-treated with the CCl_4_ + plain LPHNPs formula revealed focal hepatocellular necrosis and apoptosis associated with inflammatory cell infiltration as well as ballooning degeneration of hepatocytes (Fig. 4d). On the other hand, moderate amelioration of the histopathological alterations was recorded for the livers of rats co-treated with CCl_4_ + BSS-LPHNPs (200 mg/kg). The inspected sections from this group showed ballooning degeneration of some hepatocytes and cytoplasmic vacuolization and microvesicular steatosis of other hepatocytes (Fig. 4e). Additionally, there was marked regression of the histopathological lesions in the liver of rats co-treated with CCl_4_ + BSS-LPHNPs (400 mg/kg). Most examined sections showed that the histology of hepatic parenchyma was restored. Some examined sections from this group showed necrosis of sporadic hepatocytes (Fig. 4f). Figure (5) summarizes the histopathological lesions score in different groups, which were higher for the groups treated with CCl_4_, CCl_4_ + BSS and CCl_4_ + plain LPHNPs than for the control group. Amelioration of the lesions score was noted in the group treated with CCl_4_ + BSS-LPHNPs (200 mg/kg). High restoration of the histopathological lesions score was recorded in the liver of rats co-treated with CCl_4_ + BSS-LPHNPs (400 mg/kg).

### Immunohistochemical analysis (Cleaved caspase-3 expression)

**Figure 6:**
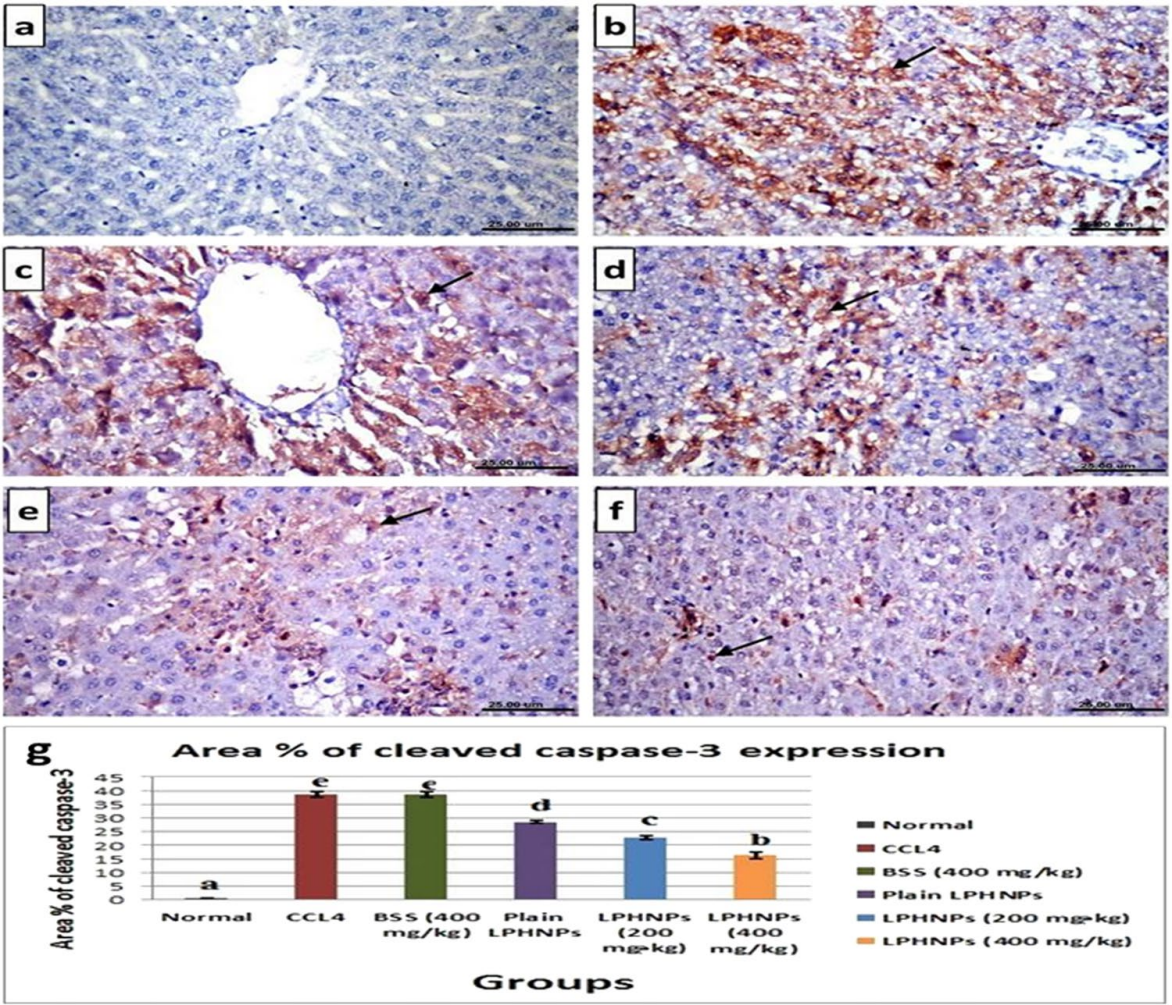
Immunostaining for cleaved caspase-3 protein in liver sections of rats, (**a**) normal control group showing no cleaved caspase-3 immune-reactive cells. (**b**) Treated with CCl_4_ showing strong positive immune expression (arrow). (**c**) Co-treated with CCl_4_ + BSS suspension showing strong positive immune reactive hepatocytes (arrow). (**d**) Co-treated with CCl_4_ + plain LPHNPs formula, showing moderate positive immune expression (arrow). (**e**) Co-treated with CCl_4_ + BSS-LPHNPs (200 mg/kg), (**f**) co-treated with CCl_4_ + BSS-LPHNPs (400 mg/kg), (**e,f**) showing weak positive cleaved caspase-3 reaction (arrow). (**g**) Immunostaining area (%) of cleaved caspase-3 expression. Data shown as mean ± SE; error bars show the variations of determinations in terms of standard errors, mean values with unlike superscript letters were significantly different(p ≤ 0.05).

Immunohistochemical analysis revealed no cleaved caspase-3 immune-reactive hepatocytes in liver sections of normal control rats (Fig. 6a). On contrary, strong positive cleaved caspase-3 immune expression was noticed in examined sections from groups treated with CCl_4_ as well as co-treated with CCl_4_ + BSS suspension (Fig. 6b,c). Meanwhile, livers of rats co-treated with CCl_4_ + plain LPHNPs formula, showed moderate positive immune expression (Fig. 6d). On the other hand, weak positive cleaved caspase-3 reaction was recorded in sections from groups co-treated with CCl_4_ + BSS-LPHNPs (200 mg/kg) and (400 mg/kg) respectively (Fig. 6e,f). Figure 6g revealed the evaluation of immunostaining expression of cleaved caspase-3 in rats from different experimental groups.

## Discussion

BSS-LPHNPs were prepared by the nanoprecipitation method which was reported to be reproducible, predictable and potentially suitable for scale-up^52^. BSS is a water insoluble hydrophobic drug with a log P value of 9.3^53,54^, making its formulation and delivery with acceptable bioavailability a difficult challenge^55^. Hydrophobic drugs usually have poor bioavailability when administered *in vivo* and cannot be used in their free form^56^. In our study, BSS was successfully encapsulated into LPHNPs due to the hydrophobic nature of the polymer core, which can enable the simple encapsulation and delivery of hydrophobic drugs^57^. PVA was used in all formulations as a stabilizing agent for the formulation and to help position the lipid shell layer onto the polymer core^35^.

The increase in the EE% by increasing the lipid ratio can be related to the ability of the lipid layer to act as a molecular barrier that traps the drug during encapsulation. High amounts of lipids allow formation of a thicker shell around the polymer core that can prevent the escape of the drug from droplets of the emulsion during the solidification step. Hence, an increase in the lipid concentration results in an increase in the EE% of LPHNPs^4^. Additionally, DSPE-PEG-2000, a lipid component, was reported to have the ability to reduce the leakage of encapsulated drugs, which increases the EE%^58^. On the other side, decrease in the EE% with further increase in the lipid ratio may be related to the ability of high amounts of the lipid component to compete with the hydrophobic drug to be encapsulated into the core polymer, resulting in low drug EE%^59^.

EE% increase with increase in the BSS ratio is in accordance with those finding by Balakrishnan *et al*. and Bagheri *et al*. who reported that an increase in the initial drug loading of a water insoluble drug during encapsulation increases its EE% because of the resulting driving force for the drug to be encapsulated^60,61^.

In general, for oral administration of NPs, a particle size below 300 nm is favorable as the particles can reach the microcirculation via the blood capillaries or penetrate through the pores present in the cell membranes^62,63^. Decrease of the particle size of the prepared NPs with increase in the lipid ratio is related to the lipophilic character of the lipid and its limited mobility in the aqueous phase, causing it to stay mainly on the polymeric particle surface, preventing the particles from aggregating. Increasing concentrations of DSPE-PEG-2000 provides additional surface area and a good electrical barrier to the surface that promotes size reduction^64,65^. The low lipid amount relative to that of the polymer in the formulations F1 and F4 led to the inability to form compact shells around the core polymer, resulting in a loose particle structure with a large particle size regardless of the amount of drug incorporated.

The PDI value usually reflects the quality of the dispersion; most researchers recognize that PDI values of 0.3 are optimum values; however, values of 0.5 are also acceptable^66^. In general, all formulations prepared at a high drug loading ratio have PDI values below 0.3, reflecting the high effectiveness and robustness of the preparation methods and conditions.

Zeta potential plays major role in the physical stability of a formulation because it is an indicator of the repulsion degree between particles of the same charge and is responsible for the repulsion forces between particles that inhibit particle aggregation. Therefore, a high zeta potential is desirable for better physical stability^29^.

All the prepared formulations exhibited controlled drug release, which is mainly related to drug encapsulation into the polymeric matrix (PLGA) which degrades slowly^65,67^. The faster and higher drug release from formulations prepared with the higher BSS ratio may be related to the contribution of the high drug loading to the encapsulation of the drug into the lipid layer as well as the polymer core; when the lipid layer dissolves in PBS (pH 7.4) faster than does the polymer core, the drug content is released causing high initial drug release from these formulations. It also may be related to the high BSS EE% of the formulations prepared at higher BSS ratio (F4, F5 and F6) compared to those prepared at lower BSS ratio (F1, F2 and F3).

Some of the essential parameters for vesicular drug delivery formulations are the optimization of physical and chemical stability and storage conditions, especially for LPHNPs, due to the possibility of drug leaching and escape from the polymer, consisting the core, and the lipid, consisting the coat, which can affect the EE% of the formulation or due to possible particles aggregation that can increase the particle size and PDI of the formulation.

The efficacy of a hepatoprotective drug can be estimated by its ability to reduce the destructive effects and maintain the normal liver physiological mechanisms that were imbalanced by hepatotoxic inducers^68^. ALT and AST enzymes are highly concentrated in the liver and kidney cells, so their elevated levels in the serum are indication of cellular leakage, and ruptured and leaky cell membranes with loss of functional integrity of the liver cell membrane^69,70^. MDA level increase and CAT level decrease are indicative of a high level of lipid peroxidation, damage and alterations of the cellular membrane structure and function resulting in absence of a defense mechanisms to prevent the free radicals formation^71^. An increase in bilirubin concentration either in the serum or the tissue gives indication about the obstruction in the bile excretion which may be attributed to liver damage^68^. All these dramatic changes occurred in different liver enzymes of rats after CCl_4_ injection emphasized the hepatic injury and occurrence of the CCl_4_ hepatotoxic effect. CCl_4_ injection didn’t significantly affect the TP levels in all groups which may be related to the fact that the TP levels may require chronic not acute treatment with CCl_4_.

Pre-treatment of rats with BSS-LPHNPs at a dose of 400 mg/kg could restore the liver enzymes (ALT and AST), liver lipid peroxidation markers (MDA and CAT), T. Bil and ALB to their normal levels after hepatotoxicity induction in rats by CCl_4_ which indicates that this formulation with the given dose regimen could protect the liver against acute hepatotoxicity induced by CCl_4_.

CYP2E1 is known to mediates the hepatocyte damage caused by CCl_4_ as the latter is metabolized in the liver by CYP2E1 yielding free radicals which results in liver injury^72^. In this study, It was mandatory to investigate if the prepared BSS-LPHNPs, at the higher dose (400 mg/Kg), have an inhibitory effect on CYP2E1 enzyme or not. Non significant change of the CYP2E1 activity after treatment with BSS-LPHNPs formulation was found indicating that BSS in this formulation has no inhibitory effect on CYP2E1 enzyme activity and emphasizing that CCl_4_ was metabolized in the liver. On the other side, significant decrease of the enzyme activity after treatment the liver microsomes with chlormethiazole which is known to be CYP2E1 inhibitor gives an indication of the assay method validity to measure the enzyme activity.

Histopathological and immunohistochemical studies confirmed that BSS-LPHNPs, mostly in the higher dose (400 mg/Kg), can act as a hepatoprotective formulation as it could restore most of the normal histological features of the hepatic parenchyma and decrease the cleaved caspase-3 expression. CCl_4_ affects the plasma membrane and destroys the phospholipid bilayer in mitochondria^73^, which initiates caspase-3-dependent apoptosis. Caspase-3 is one of the main death proteases which catalyses the specific cleavage of key cellular proteins^74^. In our study, over expression of cleaved caspase-3 specified enhanced apoptosis in the rats livers after CCl_4_ injection. However, treatments with BSS-LPHNPs significantly decreased the expression of active caspase-3 by different degrees depending on the given dose.

The moderate positive immune expression showed by livers of rats co-treated with CCl_4_ + plain LPHNPs formula may be related to the previously reported assumption that lipids with their fatty acids content may be easily incorporated into the liver cells membrane resulting in normalizing the membrane tied enzymes activity and reducing the cell injury^75^ which led to decreasing the immune expression by livers of rats treated with the plain formulation containing DSPE-PEG-2000 lipid.

These results indicate the importance of formulating BSS into a suitable delivery system to enhance its absorption from the intestinal membrane to the systemic circulation and then to the target organ, especially because free BSS is hardly absorbed into the plasma through the small intestine^76^. That is why there was usually a highly significant difference in all measured liver enzymes and markers level between the BSS-LPHNPs (400 mg/kg) and BSS free drug at the same dose. In a previous study, using another delivery system, liposomes, BSS was poorly absorbed from the small intestine and liposomalization could not enhance the low absorption of the drug^55^.

## Conclusion

BSS was successfully isolated from *Centaurea pumilio* L. and identified. Using PLGA as a polymer and DSPE-PEG-2000 as a lipid component, BSS was formulated as LPHNPs at different lipid and drug ratios. The prepared BSS-LPHNPs formulation with a 2:2:2 lipid: polymer: BSS ratio (F5) had the highest EE% and drug release with acceptable particle size, PDI and zeta potential values. This formulation showed good stability up to 90 days when stored at 4 °C/60 ± 5% RH, and significant instability from the day 15 when stored at 25 °C/60 ± 5% RH. When evaluated *in-vivo* for its hepatoprotective effect against CCl_4_ induced-hepatotoxicity, BSS-LPHNPs (F5) formulation at a dose of 400 mg/kg, without affecting the CYP2E1 activity, was able to restore the liver enzymes (ALT and AST), liver lipid peroxidation markers (MDA and CAT), T. Bil and ALB to their normal levels. In addition, it maintained the normal histological structure of the liver tissues and decreased the caspase-3 cleavage after CCl_4_- hepatotoxicity induction in rats.
